# Cardiovascular-Kidney-Metabolic Effects: Steroidal and Nonsteroidal Mineralocorticoid Receptor Antagonists

**DOI:** 10.31083/RCM38690

**Published:** 2025-07-29

**Authors:** Biykem Bozkurt, James L. Januzzi, Shweta Bansal

**Affiliations:** ^1^Winters Center for Heart Failure Research, Cardiovascular Research Institute, Baylor College of Medicine, Houston, TX 77030, USA; ^2^Cardiology Division, Massachusetts General Hospital and Harvard Medical School, Boston, MA 02115, USA; ^3^Baim Institute for Clinical Research, Boston, MA 02215, USA; ^4^Division of Nephrology, Department of Medicine, University of Texas Health San Antonio, San Antonio, TX 78229, USA

**Keywords:** cardio-kidney-metabolic syndrome, heart failure, mineralocorticoid receptor antagonists

## Abstract

Cardiovascular (CV)-kidney-metabolic (CKM) syndrome is a complex disorder characterized by the co-occurrence of CV risk factors, including chronic kidney disease (CKD), hypertension, and metabolic dysfunction, which creates a vicious cycle where one factor negatively impacts the others, ultimately leading to poor overall CV and kidney outcomes. Overactivation of the mineralocorticoid receptor, through binding with aldosterone and ligand-independent mechanisms, is implicated in the pathogenesis of CKM; mineralocorticoid receptor antagonists (MRAs) can block this interaction. Steroidal MRAs are currently recommended for people with heart failure (HF) with reduced ejection fraction and hypertension; however, the role of nonsteroidal MRAs in CKM is evolving. Indeed, steroidal MRAs have demonstrated efficacy against composite CV-related mortality and hospitalization, elevated systolic blood pressure, and hospitalizations for worsening HF in clinical trials of individuals with HF, CKD, and treatment-resistant hypertension. Moreover, the nonsteroidal MRA finerenone has demonstrated risk reductions for composite CV-related outcomes and CKD progression in patients with HF with mildly reduced or preserved ejection fraction and people with CKD associated with type 2 diabetes. Ongoing phase 3 trials are evaluating the efficacy and safety of nonsteroidal MRAs in individuals with HF and reduced ejection fraction, as well as those with mildly reduced or preserved ejection fraction, potentially expanding their role in managing CKM conditions. This review examines current clinical evidence for the use of MRAs in people with CKM syndrome.

## 1. Introduction

In 2021, cardiovascular (CV) disease (CVD) was prevalent in approximately 620 million people globally, including around 57 million cases of heart failure (HF) [1, 2]. Metabolic factors (such as obesity and diabetes) are known to predispose individuals to CVD and HF and a bidirectional association exists between chronic kidney disease (CKD) and CVD, referred to as chronic CV-kidney disorder; the interplay of these conditions is referred to as CV-kidney-metabolic (CKM) syndrome [3, 4]. CKM syndrome is therefore defined as a systemic disorder characterized by pathophysiological interactions among metabolic risk factors, CKD, and CVD, including HF. The clinical implications are significant as poor CKM health leads to multiorgan dysfunction and a high rate of adverse CV outcomes [3].

The intersection of CKM conditions is common, particularly in people with HF [5]. An analysis of five randomized clinical trials (RCTs) in HF with preserved ejection fraction (HFpEF) or mildly reduced ejection fraction (HFmrEF) demonstrated a substantial prevalence of CKM conditions in trial participants at baseline [5]. Notably, there was a greater proportion of study participants with more than one CKM condition in contemporary trials compared with those enrolled in older trials, indicating an increase in the prevalence of CKM conditions over time [5]. The elevated presence of CKM multimorbidity in recent contemporary HF trials was predominantly driven by an increased prevalence of diabetes mellitus and CKD [5]. These findings, in addition to other studies demonstrating a global increase in CVD, CKD, and diabetes, underscore the need for therapies that can target simultaneous CKM pathways in multimorbid populations, particularly HF [6, 7, 8].

The overlapping pathophysiologic mechanisms associated with CKM are in part driven by activation of the renin-angiotensin-aldosterone system, which regulates blood pressure, fluid balance, and tissue homeostasis [9, 10]. Aldosterone is an effector hormone in this pathway, exerting its effect through binding to mineralocorticoid receptors (MRs). Overactivation of these receptors can lead to inflammation and fibrosis, contributing to the progression of CV and kidney disease [11, 12, 13]. The MR has therefore emerged as a key therapeutic target, with the effects of MR antagonism showing beneficial results in completed studies [14, 15, 16]. Ongoing trials aim to explore this drug class further across the spectrum of CKM conditions, including HF. This review discusses the current clinical evidence on steroidal and nonsteroidal MR antagonists (MRAs) across CKM conditions and provides a rationale for assessing the effects of the nonsteroidal MRA, finerenone, in people with HF and CKD.

## 2. The Role of Mineralocorticoid Receptor Overactivation in Cardiovascular-Kidney-Metabolic Syndrome

The MR is a nuclear receptor expressed in the kidney, heart, and fibroblasts, as well as in the colon, brain, skin, lungs, liver, skeletal muscle, and salivary and sweat glands [12, 17, 18]. The MR controls sodium reabsorption and potassium secretion, thereby regulating blood pressure and fluid homeostasis [9, 19]. Aldosterone, a steroid hormone primarily produced by the adrenal glands, is a major ligand for the MR [9, 11].

In CKD and HF, the MR is overactivated, and this may be attributable to factors such as increased aldosterone release, ligand-independent activation from hyperglycemia, high salt load, and obesity-induced generation of reactive oxygen species [12, 20, 21]. Experimental models have suggested that overactivation of the MR can contribute to CV damage [22], increased salt and water retention, and the expression of target genes related to inflammatory and fibrotic pathways [12, 19, 23, 24, 25]. In addition, MR activation can stimulate apoptosis and cause vasoconstriction in the heart and kidneys [9]. The combined effects of MR overactivation can lead to organ injury and end-stage kidney failure [26, 27]. Overall, the results from preclinical studies implicate MR as an appropriate therapeutic target for CKD and CVD.

### Mode of Action of Steroidal and Nonsteroidal MRAs

**Fig. 1.**
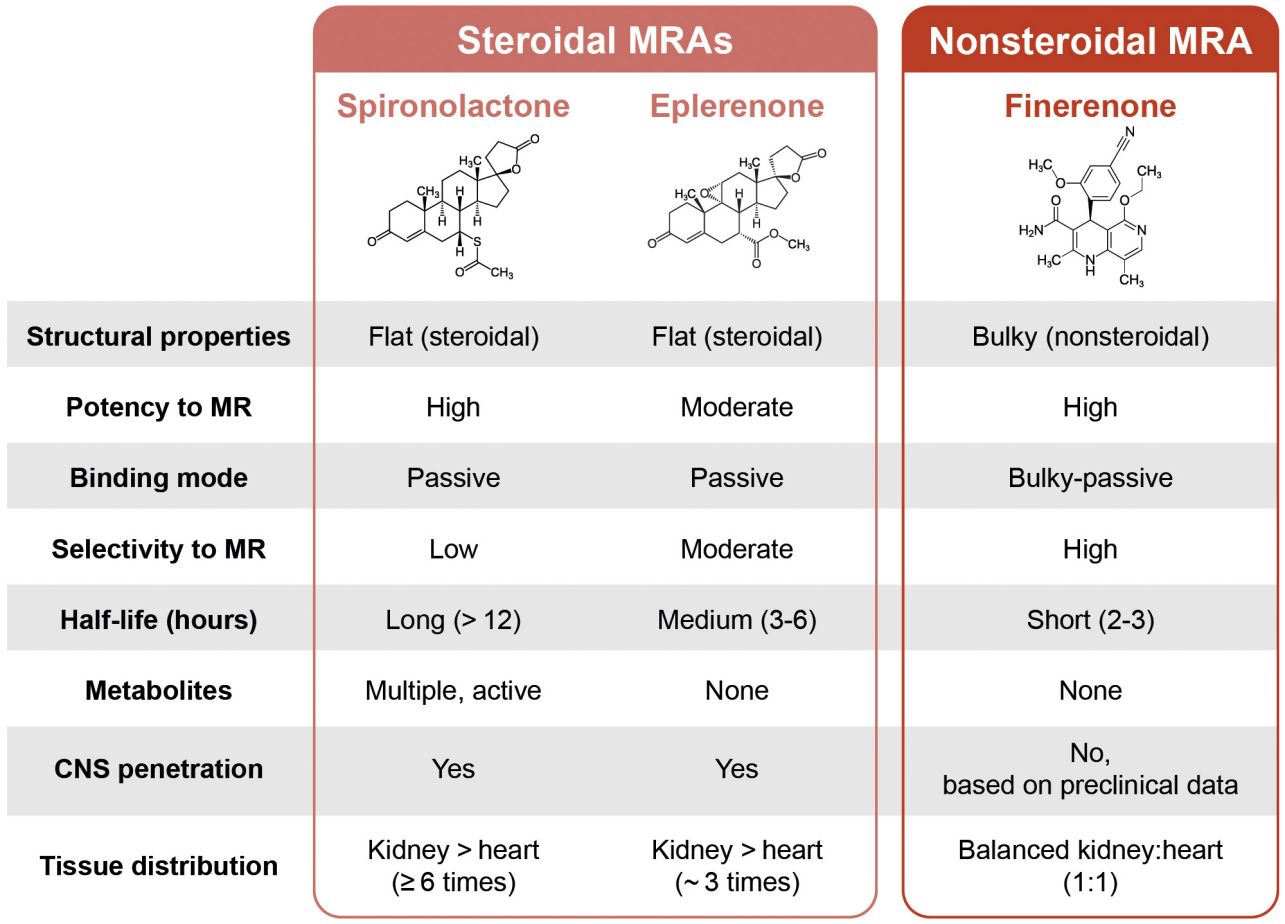
**The structural similarities and differences between steroidal and nonsteroidal mineralocorticoid receptor antagonists**. CNS, central nervous system; MR, mineralocorticoid receptor; MRA, mineralocorticoid receptor antagonist.

Aldosterone binding to MR results in translocation into the nuclear compartment, where MR binds as a dimer to MR-specific response elements and recruits transcriptional coregulators, allowing the transcription or repression of its target genes [28]. MR activation can be managed with the use of MRAs, which competitively inhibit aldosterone binding. At present, the MRA class includes agents that are steroidal in structure (such as spironolactone and eplerenone) and nonsteroidal (such as finerenone). The structural characteristics of steroidal MRAs and finerenone are described in Fig. 1. Steroidal MRAs are similar to aldosterone, with a steroid backbone and a ‘flat’ structure. In contrast, nonsteroidal MRAs do not have a steroid backbone and are described as ‘bulky’. This allows distinct and specific contact in the binding pocket of the MR, resulting in greater selectivity and potency compared with steroidal MRAs, and differential recruitment of co-regulators to give a more targeted anti-inflammatory and anti-fibrotic gene expression profile [12, 19, 28, 29, 30].

An *in vitro* study demonstrated that finerenone was associated with a greater potency and selectivity for the MR compared with steroidal MRAs spironolactone and eplerenone (half-maximal inhibitory concentration: 17.8 vs 24.2 vs 990 nM, respectively); finerenone also has very high selectivity for the MR compared with the glucocorticoid receptor, androgen receptor, and progesterone receptor (>500-fold) [31, 32]. A biomarker sub-study of the phase 3 FIGARO-DKD trial indicated that finerenone modulates several proteins involved in hemodynamic control and fibrosis, potentially contributing to clinical benefits [33].

Furthermore, as demonstrated by whole-body autoradiography in rats, the tissue distribution of finerenone was similar between the heart and kidneys (4409 vs 3782 µg-eq/L, respectively) [34], while eplerenone and spironolactone were found to be enriched in the kidneys (kidney:heart tissue distribution ratios: ≥6:1 and 3:1 for spironolactone and eplerenone, respectively) [26].

Several preclinical studies have compared the effects of finerenone versus steroidal MRAs in animal models of heart and kidney disease. In a transverse aortic constriction model, finerenone and eplerenone led to differential gene expression profiles in the hearts of treated animals [35]. In a rodent model of isoproterenol-induced cardiac fibrosis, finerenone inhibited expression of profibrotic *tenascin-X* in cardiac myocytes, while equinatriuretic doses of eplerenone did not [30]. In the same model, finerenone was more potent in inhibiting cardiac accumulation of collagen fibers and macrophage infiltration than eplerenone [30]. In a chronic model of hyperaldosteronism-induced end-organ injury, finerenone reduced renal expression of various pro-inflammatory and profibrotic biomarkers more efficiently than equinatriuretic doses of eplerenone [34]. These results suggest the mechanism of MR modulation with finerenone is distinct to that of steroidal MRAs.

## 3. Efficacy and Safety of Mineralocorticoid Receptor Antagonists in Heart Failure

### 3.1 Efficacy in the Prevention of HF

Optimal treatment of hypertension and other comorbidities may be associated with prevention of HF, but the role of steroidal MRAs in reducing new-onset HF has not been extensively investigated in large-scale clinical trials. In the CLEAR SYNERGY (OASIS-9 [NCT03048825]) trial in adults with myocardial infarction, spironolactone reduced the risk of new or worsening HF observed in 58 patients (1.6%) in the spironolactone group compared with 84 patients (2.4%) in the placebo group (hazard ratio [HR] 0.69, 95% confidence interval [CI] 0.49–0.96) [36]. However, after adjusting for the competing risk of death, the association was less definitive (HR 0.77, 95% CI 0.51–1.16) [36]. This and other clinical studies discussed are summarized in Table 1 (Ref. [36, 37, 38, 39, 40, 41, 42, 43, 44, 45, 46, 47, 48, 49, 50]).

**Table 1.** **Cardiovascular outcomes in studies investigating mineralocorticoid receptor antagonists**.

Trial (NCT)	Population (*N* randomized)	Active treatment (*n*)	Comparator (*n*)	CV-related outcomes
CLEAR SYNERGY; OASIS-9 (NCT03048825) [36]	Myocardial infarction (7062)	Spironolactone (3537)	Placebo (3525)	New or worsening HF (HR vs placebo [95% CI]): 0.69 (0.49–0.96)
				New or worsening HF, adjusted for competing risk of death by non-CV causes (HR vs placebo [95% CI]): 0.77 (0.51–1.16)
RALES [38]	Severe HF and LVEF no more than 35% (1663)	Spironolactone (822)	Placebo (841)	All-cause death (RR vs placebo [95% CI]): 0.70 (0.60–0.82)
				Hospitalization for worsening HF (RR vs placebo [95% CI]): 0.65 (0.54–0.77)
ALDO-DHF [39]	HFpEF (LVEF ≥45%) (422)	Spironolactone (213)	Placebo (209)	NT-proBNP (GMT [95% CI]): 0.86 (0.75–0.99)
				Placebo-adjusted decline of left ventricular mass index from baseline to 12 months (95% CI): –6 g/m^2^ (–10 to –1)
TOPCAT (NCT00094302) [40]	HFpEF (3445)	Spironolactone (1722)	Placebo (1723)	Composite of CV-related death, HF hospitalization, and aborted cardiac arrest (HR vs placebo [95% CI]): 0.89 (0.77–1.04)
EPHESUS [41]	Left ventricular dysfunction after myocardial infarction (6642)	Eplerenone (3319)	Placebo (3313)	CV-related death or hospitalization (RR vs placebo [95% CI]): 0.87 (0.79–0.95)
EMPHASIS-HF (NCT00232180) [42]	Chronic systolic HF (2737)	Eplerenone (1364)	Placebo (1373)	CV-related death or hospitalization for HF (HR vs placebo [95% CI]): 0.63 (0.54–0.74)
AMBER (NCT03071263) [43]	Uncontrolled-resistant hypertension and CKD (295)	Spironolactone + placebo (148)	Spironolactone + patiromer (147)	-
ARTS [44]	HFrEF with mild/moderate CKD (458)	Finerenone: 2.5 mg qd (82), 5 mg qd (83), 10 mg qd (84), 5 mg bid (64)	Spironolactone (25 mg uptitrated to 50 mg) (63)	Median change of NT-proBNP at day 15 (pg/mL [IQR]):
				Placebo: 8.2 (–373 to 263)
			Placebo (81)	Finerenone: 2.5, 5, 10 mg qd, 5 mg bid = –11.8 (–311 to 322), –60.0 (–424 to 40), –182.2 (–737 to –28), –64.4 (–466 to 59), respectively
				Spironolactone: –71.2 (–746 to 104)
				Median change of NT-proBNP at day 29 (pg/mL [IQR]):
				Placebo: 23.8 (–466 to 415)
				Finerenone: 2.5, 5, 10 mg qd, 5 mg bid = 31.3 (–426 to 348), –3.4 (–364 to 186), –193.7 (–630 to 102), –106.4 (–581 to 147), respectively
				Spironolactone: –170.3 (–585 to 70)
ARTS-HF (NCT01807221) [45]	Worsening chronic HFrEF with T2D and/or CKD (1066)	Finerenone (initial dose to uptitrated dose): 2.5–5 mg (172), 5–10 mg (163), 7.5–15 mg (167), 10–20 mg (169), 15–20 mg (163)	Eplerenone 25–50 mg (221)	Proportion of patients with >30% decrease of NT-proBNP from baseline at day 90 (%):
				Finerenone: 2.5–5 mg = 30.9, 5–10 mg = 32.5, 7.5–15 mg = 37.3, 10–20 mg = 38.8, 15–20 mg = 34.2
				Eplerenone: 37.2
				Risk of patients experiencing composite endpoint of death from any cause, CV hospitalizations, or emergency presentation for worsening chronic HF at day 90 (HR versus eplerenone [95% CI]):
				Finerenone: 2.5–5 mg = 1.21 (0.82–1.79), 5–10 mg = 0.70 (0.44–1.10), 7.5–15 mg = 0.77 (0.50–1.19), 10–20 mg = 0.56 (0.35–0.90), 15–20 mg: 0.89 (0.58–1.36)
Comparative post-hoc analysis of FIDELITY and AMBER subgroup cohorts [46]	TRH and CKD (FIDELITY: 624; AMBER: 295)	FIDELITY: finerenone (316)	FIDELITY: placebo (308)	LS mean group difference of change in SBP from baseline between intervention and comparator (mmHg):
		AMBER: spironolactone + patiromer (147)	AMBER: spironolactone + placebo (148)	FIDELITY (at ∼17 weeks): –5.74 (95% CI –7.99 to –3.49); *p * < 0.0001
				AMBER (at 12 weeks): –1.0 (95% CI –4.4 to 2.4); *p* = 0.58
FIDELIO-DKD (NCT02540993) [47]	T2D and CKD, treated with ACEi or ARB at maximum tolerated dose (5734)	Finerenone (2833)	Placebo (2841)	Composite CV outcome of CV-related death, nonfatal myocardial infarction, nonfatal stroke, or hospitalization for HF (HR [95% CI]): 0.86 (0.75–0.99)
FIGARO-DKD (NCT02545049) [37]	T2D and CKD, treated with ACEi or ARB at maximum tolerated dose (7437)	Finerenone (3686)	Placebo (3666)	Composite CV outcome of CV-related death, nonfatal myocardial infarction, nonfatal stroke, or hospitalization for HF (HR [95% CI]): 0.87 (0.76–0.98)
				Hospitalization for HF (HR [95% CI]): 0.71 (0.56–0.90)
FIDELITY (pooled analysis of FIDELIO-DKD and FIGARO-DKD) [48]	T2D and CKD, treated with ACEi or ARB at maximum tolerated dose (13,026)	Finerenone (6519)	Placebo (6507)	Composite CV outcome of CV-related death, nonfatal myocardial infarction, nonfatal stroke, or hospitalization for HF (HR [95% CI]): 0.86 (0.78–0.95)
				Hospitalization for HF (HR [95% CI]): 0.78 (0.66–0.92)
FINEARTS-HF (NCT04435626) [49]	HF with an LVEF ≥40% (6016)	Finerenone (3003)	Placebo (2998)	Total worsening HF events and death from CV causes (RR [95% CI]): 0.84 (0.74–0.95)
				Total worsening HF events (RR [95% CI]): 0.82 (0.71–0.94)
FINE-HEART (pooled analysis of FIDELIO-DKD, FIGARO-DKD, and FINEARTS-HF) [50]	CKM conditions; HF with an LVEF ≥40%; or CKD with T2D (18,991)	Finerenone (9501)	Placebo (9490)	CV death and hospitalization for HF (HR [95% CI]): 0.85 (0.78–0.93)
				Major adverse CV events (HR [95% CI]): 0.91 (0.85–0.98)
				HF hospitalization (HR [95% CI]): 0.83 (0.75–0.92)

ACEi, angiotensin-converting enzyme inhibitor; ARB, angiotensin receptor blocker; bid, twice daily; CI, confidence interval; CKD, chronic kidney disease; CKM, cardiovascular-kidney-metabolic; CV, cardiovascular; GMT, geometric mean titer; HF, heart failure; HFmrEF, heart failure with mildly reduced ejection fraction; HFpEF, heart failure with preserved ejection fraction; HFrEF; heart failure with reduced ejection fraction; HR, hazard ratio; IQR, interquartile range; LS, least squares; LVEF, left ventricular ejection fraction; MRA, mineralocorticoid receptor agonist; NT-proBNP, N-terminal pro-brain-type natriuretic peptide; qd, once daily; RR, risk ratio; SBP, systolic blood pressure; T2D, type 2 diabetes; TRH, treatment-resistant hypertension; NCT, national clinical trial.

The nonsteroidal MRA finerenone has been studied in clinical trials that enrolled people with and without HF, enabling interrogation of new-onset HF as an endpoint. In the FIGARO-DKD study of individuals with proteinuric CKD and type 2 diabetes (T2D), finerenone treatment reduced new-onset HF by 32% in participants without a history of HF at baseline [16]. In addition, the overall effects of finerenone on reducing HF-related outcomes were not modified by baseline history of HF [16]. The incidence of treatment-emergent adverse events across the finerenone and placebo treatment groups was well balanced in the overall population and in the subgroup of study participants without HF at baseline [16, 37]. Finerenone was associated with a greater incidence of hyperkalemia compared with placebo (11.0 vs 5.3%, respectively) in participants without HF at baseline; however, only 1.3% led to finerenone treatment discontinuation [16].

### 3.2 Clinical Evidence of the Prevention of CV Death in People With HF

As shown in Table 1, clinical studies have demonstrated that steroidal and nonsteroidal MRAs are both associated with a reduction in the risk of composite CV outcomes (which often include CV death and HF hospitalizations) in patients with HF. The outcome component of CV death is of particular interest as 1- and 5-year survival rates in people with worsening HF are low, less than or comparable to those observed with some forms of cancer [51, 52]. Therefore, gains in event-free survival have significant impact in the setting of HF, particularly multimorbid HF. Individual HF trials are often not sufficiently powered to examine treatment effects on the endpoint of CV death alone; therefore, pooled analyses have been performed to better inform the potential CV mortality risk reduction with MRAs. In a pooled meta-analysis of four HF trials with MRAs (RALES, EMPHASIS-HF, TOPCAT, and FINEARTS-HF; *N* = 13,846), steroidal and nonsteroidal MRAs reduced CV death by 19% with a possibly greater treatment effect seen in trials of heart failure with reduced ejection fraction (HFrEF) compared with HFmrEF or HFpEF (*p*_interaction_ = 0.082) [15].

FINE-HEART, a prespecified pooled analysis (*N* = 18,991) of three phase 3 RCTs of finerenone in people with CKD and T2D (FIDELITY program) or HFmrEF and HFpEF (FINEARTS-HF) assessed the effect of finerenone on reducing CV death [50]. In this analysis, the primary outcome of CV death occurred in 4.4 and 5.0% of study participants randomized to finerenone and placebo, respectively; this difference was not statistically significant [50]. However, finerenone was associated with reductions in all-cause mortality and CV deaths when combined with deaths of indeterminate cause in a sensitivity analysis [50]. Notably, the treatment effects of finerenone were generally consistent across subgroups, including those representing the spectrum of CKM conditions. These data therefore support the disease-modifying potential of finerenone in broad, high-risk patient populations, although certain ethnic populations were underrepresented in these trials and greater inclusion of Black participants would be warranted in future studies.

### 3.3 Clinical Evaluation in the Treatment of People With Established HF

Steroidal MRAs are associated with reductions in CV death, all-cause mortality, and cardiac hospitalizations in people with HFrEF [53]. Individual trial data for MRA RCTs of interest are presented in Table 1.

While steroidal MRAs are associated with a reduction in the risk of CV outcomes in people with HF, this effect is less obvious in those with more severe CKD. In a pooled meta-analysis of the RALES, EMPHASIS-HF, TOPCAT, and EPHESUS trials, the treatment effect on the composite outcome of CV death and or HF hospitalization was attenuated with decreasing estimated glomerular filtration rate (eGFR) [54]. Regarding evidence for nonsteroidal MRAs in people with HF, a subanalysis of the FIDELIO-DKD study assessed the efficacy of finerenone against the key endpoints in subgroups of interest, including in adults with a history of HF at baseline. The treatment effects of finerenone in reducing the composite CV outcome (CV-related death, nonfatal myocardial infarction, nonfatal stroke, and hospitalization for HF) and the composite kidney outcome (kidney failure, a sustained decrease of at least 40% in the eGFR from baseline or death from renal causes) were consistent in participants with a history of HF at baseline [55].

Recently, the CV efficacy of finerenone was investigated in adults with HF. The FINEARTS-HF (NCT04435626) phase 3 RCT investigated the efficacy and safety of finerenone or matching placebo in reducing CV death and HF-related events across 6001 adults with chronic HFpEF (EF ≥50%) or HFmrEF (EF ≥40 and <50%) [56]. Finerenone significantly reduced the risk of the primary composite outcome (total HF events and death from CV causes) by 16% compared with placebo [56]. Efficacy outcomes were not modified by baseline disease characteristics, including eGFR category, urine albumin-to-creatinine ratio (UACR) category, presence of diabetes, and body mass index [56]. The absence of treatment effect modification by kidney function with finerenone was in contrast to the results of the pooled meta-analysis of the RALES, EMPHASIS-HF, TOPCAT, and EPHESUS trials with steroidal MRAs [54].

Findings from the pooled FINE-HEART analysis are also informative in assessing the effects of finerenone in a broad population, including people with HF with and without CKD and with and without T2D. In FINE-HEART, assessment of baseline CKM conditions demonstrated that these were common in the study populations, with 10.4, 77.5, and 12.1% having one, two, or three CKM conditions, respectively [50]. In FINE-HEART, finerenone significantly reduced the risk of the composite of CV death or HF hospitalization by 15% and major adverse CV events by 9% [50]. Treatment effects on CV death were generally consistent across subgroups, including in CKM subgroups.

Results from these trials demonstrate the high burden of CKM conditions in people with HF and illustrate the interdependence of CKD and HF and the need for therapies that can appropriately manage overlapping conditions.

### 3.4 Safety Profile in HF Studies

The steroidal structure of spironolactone allows for binding at steroid hormone receptors, leading to off-target adverse events including gynecomastia, and irregular menstruation [37, 57]. Safety data obtained in individual RCTs of MRAs are presented in Table 2 (Ref. [36, 37, 38, 39, 40, 41, 42, 43, 44, 45, 46, 47, 48, 49, 50]).

**Table 2.** **Summary of safety data from key studies of mineralocorticoid receptor antagonists**.

Trial (NCT)	Population (*N* randomized)	Active treatment (*n*)	Comparator (*n*)	Proportion of participants experiencing adverse events in active treatment group vs comparator group (%)
CLEAR SYNERGY; OASIS-9 (NCT03048825) [36]	Myocardial infarction (7062)	Spironolactone (3537)	Placebo (3525)	Hyperkalemia leading to treatment discontinuation: 1.1 vs 0.6
				Gynecomastia: 2.3 vs 0.5
RALES [38]	Severe HF and LVEF no more than 35% (1663)	Spironolactone (822)	Placebo (841)	Serious hyperkalemia: 2 vs 1
				Gynecomastia or breast pain in men: 10 vs 1
ALDO-DHF [39]	HFpEF (LVEF ≥45%) (422)	Spironolactone (213)	Placebo (209)	Gynecomastia: 4 vs <1
TOPCAT (NCT00094302) [40]	HFpEF (3445)	Spironolactone (1722)	Placebo (1723)	Hyperkalemia: 18.7 vs 9.1
				Gynecomastia leading to treatment discontinuation: 2.5 vs 0.3
EPHESUS [41]	Left ventricular dysfunction after myocardial infarction (6642)	Eplerenone (3319)	Placebo (3313)	Hyperkalemia: 3.4 vs 2.0
				Gynecomastia in men: 0.5 vs 0.6
				Breast pain in women: 0.1 vs 0.3
EMPHASIS-HF (NCT00232180) [42]	Chronic systolic HF (2737)	Eplerenone (1364)	Placebo (1373)	Hyperkalemia: 8.0 vs 3.7
				Gynecomastia or other breast disorders: 0.7 vs 1.0
AMBER (NCT03071263) [43]	Uncontrolled-resistant hypertension and CKD (295)	Spironolactone + placebo (148)	Spironolactone + patiromer (147)	Hyperkalemia or increased blood potassium level: 9 vs 6
				Hyperkalemia leading to treatment discontinuation: 7 vs 1
ARTS [44]	HFrEF with mild/moderate CKD (458)	Finerenone: 2.5 mg qd (82), 5 mg qd (83), 10 mg qd (84), 5 mg bid (64)	Spironolactone (25 mg uptitrated to 50 mg) (63)	Hyperkalemia/blood potassium level increase:
				Finerenone 2.5, 5, 10 mg qd, 5 mg bid = 4.5, 1.5, 4.5, and 7.8, respectively
			Placebo (81)	Spironolactone = 11.1; placebo = 1.5
ARTS-HF (NCT01807221) [45]	Worsening chronic HFrEF with T2D and/or CKD (1066)	Finerenone (initial dose to uptitrated dose): 2.5–5 mg (172), 5–10 mg (163), 7.5–15 mg (167), 10–20 mg (169), 15–20 mg (163)	Eplerenone 25–50 mg (221)	Hyperkalemia at any point post-baseline (potassium concentration ≥5.6 mmol/L): finerenone (total) = 4.3; eplerenone = 4.7
				Hyperkalemia at any point post-baseline (potassium concentration >6.0 mmol/L): finerenone (total) = 0.5; eplerenone= 0.5
Comparative post-hoc analysis of FIDELITY and AMBER subgroup cohorts [46]	TRH and CKD (FIDELITY: 624; AMBER: 295)	FIDELITY:	FIDELITY:	Proportion of participants with recorded treatment discontinuation due to hyperkalemia from baseline (%)
		Finerenone (316)	Placebo (308)	FIDELITY: finerenone (at ∼17 weeks) = 0.3; placebo (at ∼17 weeks) = 0
		AMBER:	AMBER:	AMBER (at 12 weeks): spironolactone + patiromer = 6.8; spironolactone + placebo = 23.0
		Spironolactone + patiromer (147)	Spironolactone + placebo (148)	
FIDELIO-DKD (NCT02540993) [47]	T2D and CKD, treated with ACEi or ARB at maximum tolerated dose (5734)	Finerenone (2833)	Placebo (2841)	Investigator-reported hyperkalemia: 18.3 vs 9.0
				Hyperkalemia related to trial regimen: 11.8 vs 4.8
FIGARO-DKD (NCT02545049) [37]	T2D and CKD, treated with ACEi or ARB at maximum tolerated dose (7437)	Finerenone (3686)	Placebo (3666)	Investigator-reported hyperkalemia: 10.8 vs 5.3
				Hyperkalemia related to trial regimen: 6.5 vs 3.1
FIDELITY (pooled analysis of FIDELIO-DKD and FIGARO-DKD) [48]	T2D and CKD, treated with ACEi or ARB at maximum tolerated dose (13,026)	Finerenone (6519)	Placebo (6507)	Investigator-reported hyperkalemia: 14.0 vs 6.9
				Hyperkalemia related to trial regimen: 8.8 vs 3.8
				Permanent discontinuation due to hyperkalemia: 1.7 vs 0.6
FINEARTS-HF (NCT04435626) [49]	HF with an LVEF ≥40% (6016)	Finerenone (3003)	Placebo (2998)	Investigator-reported hyperkalemia: 9.7 vs 4.2
				Hyperkalemia leading to hospitalization: 0.5 vs 0.2
FINE-HEART (pooled analysis of FIDELIO-DKD, FIGARO-DKD, and FINEARTS-HF) [50]	CKM conditions; HF with an LVEF ≥40%; or CKD with T2D (18,991)	Finerenone (9501)	Placebo (9490)	Hyperkalemia (based on laboratory measurements of potassium levels): 12.8 vs 6.2
				Hyperkalemia leading to treatment discontinuation: 1.3 vs 0.5
				Hyperkalemia leading to hospitalization: 0.8 vs 0.2
				Gynecomastia or breast hyperplasia: 0.2 vs 0.2

ACEi, angiotensin-converting enzyme inhibitor; ARB, angiotensin receptor blocker; bid, twice daily; CKD, chronic kidney disease; CKM, cardiovascular-kidney-metabolic; HF, heart failure; HFpEF, heart failure with preserved ejection fraction; HFrEF, heart failure with reduced ejection fraction; LVEF, left ventricular ejection fraction; NCT, national clinical trial; qd, once daily; T2D, type 2 diabetes; TRH, treatment-resistant hypertension.

The RALES study identified a greater proportion of participants experiencing gynecomastia or breast pain in the spironolactone treatment group compared with the placebo group (10 vs 1%, respectively) [38]. In the TOPCAT study, gynecomastia led to treatment discontinuation in 2.5% of participants in the spironolactone group compared with 0.3% in the placebo group [40]. Conversely, finerenone, which is highly specific for the MR receptor [32], is not accompanied by the risk for androgenic or progestogenic adverse effects [50]. In FINE-HEART, the incidence of gynecomastia or breast hyperplasia with finerenone was comparable with placebo (0.2% in both treatment groups) [50].

In addition to these steroidal effects, hyperkalemia is a common concern with use of MRAs, particularly in the context of advanced CKD [54]. Hyperkalemia has also been recorded in those treated with finerenone. The FINEARTS-HF study recorded a greater incidence of hyperkalemia with finerenone compared with placebo (9.7 vs 4.2%, respectively); however, only a small proportion of events led to hospitalization (0.5 vs 0.2%, respectively) [56]. Overall, the FINE-HEART analysis showed that a greater proportion of hyperkalemia events were observed with finerenone compared with placebo (12.8 vs 6.2%, respectively), with only a small proportion of these leading to treatment discontinuation (1.3 vs 0.5%, respectively) or hospitalization (0.8 vs 0.2%, respectively) [50]. There were no fatal hyperkalemia events [50].

An open question is whether the rates of hyperkalemia differ between steroidal and nonsteroidal MRAs. In a head-to-head study, finerenone was associated with a lower incidence of hyperkalemia compared with spironolactone [44]. The ARTS study was a phase 2 RCT that compared the efficacy of multiple doses of finerenone and spironolactone in people with HFrEF and mild or moderate CKD. Hyperkalemia occurred less frequently across all finerenone dose groups (2.5, 5, and 10 mg twice daily) than the spironolactone group (25 mg or 50 mg once daily; pooled finerenone vs spironolactone; 5.3 vs 12.7%, respectively; *p* = 0.048) [44].

## 4. Efficacy and Safety of Mineralocorticoid Receptor Antagonists in the Treatment of Chronic Kidney Disease 

### 4.1 Evidence of Efficacy in the Treatment and Progression of CKD

There are limited studies that have assessed the efficacy of steroidal MRAs in reducing progression of kidney disease in people with CKD. Data from HF outcomes trials with steroidal MRAs indicated no improvement in delaying progression of CKD [58, 59, 60]. Indeed, owing to a lack of long-term clinical results, steroidal MRAs are not indicated for reducing kidney disease progression [61]. In the BARACK-D study, no effect of spironolactone on CV outcomes was observed in people with stage 3b CKD, and spironolactone treatment was frequently withdrawn due to participants meeting prespecified stop criteria including hyperkalemia and decreased eGFR [62]. Eplerenone is contraindicated in people with decreased kidney function (creatinine clearance ≤30 mL/min) due to the increased risk of hyperkalemia [63].

In contrast to the paucity of evidence for improving CKD outcomes with steroidal MRAs, there are multiple clinical studies that have demonstrated the efficacy of finerenone for reducing the risk of CKD-related outcomes. The pooled FIDELITY analysis—in which all study participants had T2D and CKD—identified that finerenone was associated with significant reductions in the risk of the composite kidney outcome (defined as time to first onset of kidney failure, sustained ≥57% decrease in eGFR from baseline over ≥4 weeks, or renal death) as well as kidney failure and end-stage kidney disease (23, 16, and 20%, respectively) [48]. The authors suggest that these results highlight the importance of early treatment before CKD has progressed to improve outcomes in this patient population. Since these studies were initiated, sodium–glucose cotransporter 2 inhibitors (SGLT2is) have emerged as a key therapeutic approach and are now incorporated into guidelines for the optimal management of CKD. Analysis of the FIDELITY data has established that SGLT2i use had no impact on the risk reduction seen with finerenone [64]. In addition, the ongoing CONFIDENCE trial will provide further data on the simultaneous use of finerenone and SGLT2is [65]. Studies due to complete in 2026 and 2025 are evaluating the efficacy of finerenone on kidney outcomes in different CKD phenotypes (CKD of nondiabetic etiology in the FIND-CKD study and CKD in type 1 diabetes in the FINE-ONE study) [66, 67].

In FINEARTS-HF, finerenone did not reduce the occurrence of the composite kidney endpoint (defined as a sustained decrease in eGFR of ≥50% from baseline, sustained decline in eGFR to <15 mL/min/1.73 m^2^, initiation of dialysis or renal transplantation) versus placebo in people with HFmrEF or HFpEF [68]. The frequency of kidney composite outcome events in this population was low, occurring in only 1.8 and 2.5% of participants randomized to placebo and finerenone, respectively, indicating that study participants were at low risk for adverse kidney events [68]. The chronic eGFR slope with placebo was 1.1 mL/min/1.73 m^2^/year, comparable to the eGFR decline associated with aging [68]. Finerenone significantly reduced UACR by 30% at 6 months and reduced new-onset micro- or macroalbuminuria compared with placebo [68]. The reduction in albuminuria may be considered clinically meaningful as UACR reductions of ≥30% in 6 months correspond to reductions in “hard” kidney composite outcomes [69].

However, in the FINE-HEART pooled analysis, which included participants with and without CKD, finerenone was seen to significantly reduce the risk of the kidney composite endpoint (defined as sustained decrease in eGFR to ≥50% from baseline, sustained decline in eGFR to <15 mL/min/1.73 m^2^, kidney failure, and death due to kidney failure) by 20% compared with placebo [50].

Beyond potential impact on aldosterone-mediated tissue fibrosis, the treatment effect of finerenone on delaying CKD may also be attributed to its hemodynamic effects. This is evidenced by the acute effects of finerenone on eGFR following treatment initiation. Similar to other agents that delay CKD progression, treatment with finerenone may result in a slight eGFR drop within the first few months of treatment consequent to a decrease in glomerular hypertension and reduced hyperfiltration [47, 70, 71, 72]. Change in eGFR over time with finerenone after this acute period (chronic eGFR slope) becomes less steep compared with placebo, reflecting a beneficial effect [47, 73]. This may delay further nephron loss and slow the progression of CKD translating to reduced adverse kidney and CV events [70].

### 4.2 Safety Profile in CKD

Although the long-term impact of acute eGFR changes on CV outcomes or worsening kidney function in people with HF is not fully known, a decline in eGFR has traditionally been captured as a safety endpoint in clinical trials.

In HF outcome trials, steroidal MRAs were associated with acute eGFR declines within the first few weeks of treatment initiation [58, 74]. In RALES, decline in eGFR of ≥30% in the first 12 weeks after randomization occurred more frequently with spironolactone than placebo (17 vs 7%) [74]. In study participants who experienced an eGFR decline of ≥30%, hyperkalemia adverse events were recorded for 30.2 and 13.3% of those randomized to spironolactone and placebo, respectively [74]. In EPHESUS, eplerenone was associated with more frequent decline in eGFR of >20% compared with placebo in the first month after randomization [58]. Analyses from the TOPCAT study reported a greater frequency of at least doubling of serum creatinine to above the reference limits with spironolactone compared with placebo in adults with HFpEF [75].

The treatment effects of spironolactone and eplerenone versus placebo on efficacy outcomes in RALES, EPHESUS, and TOPCAT were not influenced by post-randomization changes in eGFR and serum creatinine [58, 74, 75]. Nevertheless, the reported effects of steroidal MRAs on renal function, typically seen in those with advanced CKD, have led to limits in the use of steroidal MRAs in clinical practice. A study investigating the predictors of MRA underuse in a cohort of individuals with HFrEF identified that a creatinine clearance of <30 and 30 to <60 mL/min was one of the factors associated with non-use of MRAs [76]. These findings indicate a clinical need in people with CKM conditions in HF.

The FIDELITY study recorded similar incidences of treatment-emergent adverse events across the finerenone and placebo intervention groups. While incidences of hyperkalemia adverse events occurred more frequently with finerenone compared with placebo (14.0 vs 6.9%), no events were fatal and a small proportion led to finerenone treatment discontinuation or hospitalization (1.7 and 0.9%, respectively) [48]. Furthermore, the FIDELIO- and FIGARO-DKD trials recorded an acute decline in eGFR over the first 4 months of finerenone treatment [47, 73]. However, the beneficial effect of finerenone on reducing the risk of both the CV and kidney composite outcomes in FIDELITY was consistent when eGFR change at 1 month was modelled as a continuous variable (*p*_interaction_ = 0.58 and 0.36, respectively) [77].

A post-hoc analysis study investigated CV outcomes in people with treatment-resistant hypertension and CKD treated with finerenone and spironolactone from the FIDELITY and AMBER (NCT03071263) RCT cohorts, respectively. The proportion of participants with recorded treatment discontinuation due to hyperkalemia was lower in the finerenone FIDELITY cohort when compared with the spironolactone + patiromer or spironolactone + placebo cohorts (0.3 vs 6.8 vs 23.0%, respectively) [46].

## 5. Efficacy of Mineralocorticoid Receptor Antagonists in the Prevention of New-Onset Diabetes

There are very few studies that have assessed the impact of steroidal MRAs and finerenone on glycemic control; of those that have, this outcome was not part of the original study design. A randomized cross-over study investigated the effects of aldosterone blockade with spironolactone in hypertensive adults with T2D [78]. Spironolactone was associated with a numerically small but statistically significant increase in glycated hemoglobin (HbA1c) of 0.21% (95% CI 0.05–0.37 [*p* = 0.01]), when compared with placebo [78].

Although the FIDELIO- and FIGARO-DKD studies enrolled participants with T2D, these studies were not designed to assess the impact of finerenone on glycemic control. In FIDELIO- and FIGARO-DKD, glycemic control was balanced between the finerenone and placebo arms throughout the duration of each trial [37, 47]. This reflects the equivalent management of diabetes irrespective of randomization, making interrogation of diabetes-related outcomes in these trials challenging. The recently published FINEARTS-HF study enabled investigation of finerenone on diabetes-related outcomes as the study enrolled people both with and without diabetes. Specifically, an analysis from the FINEARTS-HF study assessed the impact of finerenone on new-onset diabetes [79]. At baseline, approximately 60% of participants without diabetes had prediabetes, and all study participants were receiving a similar proportion of background treatments [79]. Finerenone significantly reduced the risk of new-onset diabetes (defined as HbA1c levels ≥6.5% on two consecutive follow-up visits or initiation of glucose-lowering therapies) compared with placebo. This reduction was similar regardless of the presence or absence of background SGLT2is (reductions of 24 and 26%, respectively) [79].

## 6. Mineralocorticoid Receptor Antagonists in the Treatment of Hypertension

Steroidal MRAs are recommended for treatment-resistant hypertension [80]. The PATHWAY-2 study identified that spironolactone was associated with the largest average reduction in home systolic blood pressure (SBP) compared with placebo, doxazosin, and bisoprolol with measures taken at 6 and 12 weeks post-treatment initiation in people with treatment-resistant hypertension [81]. Treatment discontinuations due to renal impairment, hyperkalemia, and gynecomastia were not increased with spironolactone relative to the comparator treatments and placebo [81]. In addition, a meta-analysis showed that aldosterone antagonists are associated with reductions in SBP and diastolic blood pressure in clinical trial participants with treatment-resistant hypertension [82]. AMBER was a phase 2 RCT that investigated the combination of spironolactone and patiromer in people with CKD and hypertension [43]. Significant reductions in automated office SBP between baseline and week 12 were recorded across both intervention groups (spironolactone + patiromer vs spironolactone + placebo) [43].

Finerenone has not been assessed in a dedicated hypertension study. Nevertheless, hemodynamic effects on blood pressure have been reported in various studies spanning CKM conditions. The ARTS-DN study of people with T2D, UACR ≥30 mg/g, and eGFR >30 mL/min/1.73 m^2^ recorded significant changes in 24-hour ambulatory blood pressure monitoring SBP from baseline to day 90 with once daily finerenone at 10 mg (–5.8 mmHg), 15 mg (–8.7 mmHg), and 20 mg (–7.4 mmHg) doses compared with placebo (+2.5 mmHg) [83].

The ARTS study assessed both finerenone and a steroidal MRA, spironolactone, on multiple outcomes, including hemodynamic-related outcomes in participants with HFrEF (left ventricular ejection fraction [LVEF] ≤40%) and mild or moderate CKD (eGFR of 30–90 mL/min/1.73 m^2^). While both agents reduced SBP, the reduction between baseline and day 29 was significantly lower in the spironolactone arm compared with all doses of finerenone [44]. In the ARTS-HF study in people with HF with T2D and/or CKD, comparable reductions in SBP from baseline to day 90 were noted with eplerenone and finerenone treatment; no dose relationship was identified with finerenone [45].

In the post-hoc analysis of the FIDELITY and AMBER RCT cohorts, in people with CKD with treatment-resistant hypertension, there was less SBP reduction with finerenone (–7.1 mmHg at approximately 17 weeks) compared with people receiving spironolactone + patiromer (–11.7 mmHg at 12 weeks) and spironolactone + placebo (–10.8 mmHg at 12 weeks) [46]. The relatively smaller reduction in SBP with finerenone may be attributed to the long half-life of spironolactone as well as the presence of active spironolactone metabolites.

Although these data demonstrate that both steroidal and nonsteroidal MRAs exert hemodynamic effects, in general, the magnitude of blood pressure lowering is less with finerenone compared with spironolactone; thus, finerenone has a different hemodynamic profile to steroidal MRAs, which may contribute towards the altered profile of these agents in CKM conditions.

## 7. Ongoing Cardiovascular Trials

Steroidal MRAs are being investigated in people with stable and symptomatic HFmrEF/HFpEF (LVEF ≥40% and New York Heart Association [NYHA] class II–IV). The ongoing SPIRRIT-HFpEF (NCT02901184) phase 3 study is investigating the effect of spironolactone (or eplerenone if spironolactone is not tolerated) on the composite incidence rate of total hospitalizations for HF or CV-related death in adults ≥50 years of age with stable HF (*N* = 2000; Table 3 (Ref. [84, 85, 86, 87, 88, 89])) [84]. This trial will assess the question of spironolactone effectiveness in the HFpEF population given the nonsignificant results on the primary endpoint in people with HFpEF shown in the TOPCAT study [40]. In addition, the SPIRIT-HF (NCT04727073) phase 3 study is investigating the effect of spironolactone on the composite of CV death and total (first and recurrent) hospitalizations for HF in adults ≥50 years of age with symptomatic HF with LVEF ≥40% (*N* = 1300; Table 3) [85].

**Table 3.** **Ongoing phase 3 heart failure outcome studies with steroidal and nonsteroidal mineralocorticoid receptor antagonists**.

Trial (NCT)	Population (*N*)	Treatment arms	Select inclusion criteria	Select exclusion criteria	Primary endpoint
SPIRRIT-HFpEF (NCT02901184) [84]	Stable HF (2000)	Spironolactone/eplerenone vs SOC	≥50 years of age	Known EF <40% ever	Incidence rate of total hospitalization for HF or CV death
			NYHA class II–IV and LVEF ≥40%; NT-proBNP >300 ng/L in SR, >750 ng/L with AF, >1200 ng/L within the last 12 m; regular use of loop diuretics		
SPIRIT-HF (NCT04727073) [85]	Symptomatic HF (1300)	Spironolactone vs placebo	≥50 years of age	Hyperkalemia 2 weeks prior to VR; hyponatremia; eGFR <30 mL/min/1.73 m^2^; serum creatinine level ≥1.8 mg/dL	Total (first or recurrent) hospitalization for HF and CV deaths
			NYHA class II–IV and LVEF ≥40%; NT-proBNP >300 pg/mL in SR, and >900 pg/mL with AF/HHF or treatment with intravenous diuretics for worsening HF		
BalanceD-HF (NCT06307652) [86]	HF (4800)	Dapagliflozin + balcinrenone vs dapagliflozin + placebo	≥18 years of age	Recent acute coronary syndrome, stroke, or transient ischemic attack; cardiac surgery within 3 months prior to enrollment; history of hypertrophic obstructive cardiomyopathy; T1D	Time to first occurrence of CV death, hospitalizations for HF, and HF event without hospitalization
			Recent HF event; NYHA class II–IV and whole-spectrum of LVEF; NT-proBNP >300 pg/mL, and >600 pg/mL if concomitant AF or atrial flutter; not taking an MRA		
REDEFINE-HF (NCT06008197) [87]	Hospitalized with HF (LVEF ≥40%) (5200)	Finerenone vs placebo	≥18 years of age	MRA treatment; history of severe hyperkalemia in the setting of MRA use; eGFR <25 mL/min/1.73 m^2^ or serum/plasma potassium >5.0 mmol/L	Composite total of HF events and CV-related death; number of AEs leading to discontinuation of study drug; number of SAEs
			Current/recent hospitalization for HF; elevated NT-proBNP ≥1000 pg/mL or BNP ≥250 pg/mL for patients without AF; or elevated NT-proBNP ≥2000 pg/mL or BNP ≥500 pg/mL for patients with AF		
CONFIRMATION-HF (NCT06024746) [88]	HF (1500)	Finerenone and empagliflozin (combination treatment) vs usual local SOC	≥18 years of age	Diagnosis of T1D/prior history of diabetic ketoacidosis; history of severe hyperkalemia in the setting of MRA use; treatment with nonsteroidal MRA or SGLT2i; eGFR <30 mL/min/1.73 m^2^ and/or serum/plasma potassium >5.0 mmol/L	Clinical benefit 6 months post-trial initiation; number of SAEs; number of AEs leading to study drug discontinuation
			Recent hospitalization for HF; elevated NT-proBNP ≥1000 pg/mL or BNP ≥250 pg/mL for patients in SR; or elevated NT-proBNP ≥2000 pg/mL or BNP ≥500 pg/mL for patients with AF; fulfilment of protocol-defined stabilization criteria; treatment with at least one intravenous dose of a loop diuretic		
FINALITY-HF (NCT06033950) [89]	HFrEF (2600)	Finerenone vs placebo	≥18 years of age	Treatment with nonsteroidal MRA; eGFR <25 mL/min/1.73 m^2^ and/or serum/plasma potassium >5.0 mmol/L	Time to first occurrence of CV death or HF event; number of SAEs; number of AEs leading to study drug discontinuation
			Symptomatic HFrEF; not on steroidal MRA		

AE, adverse event; AF, atrial fibrillation; BNP, B-type natriuretic peptide; CV, cardiovascular; EF, ejection fraction; eGFR, estimated glomerular filtration rate; HF, heart failure; HFrEF, heart failure with reduced ejection fraction; HHF, hospitalization for heart failure; LVEF, left ventricular ejection fraction; MRA, mineralocorticoid receptor agonist; NT-proBNP, N-terminal pro-brain-type natriuretic peptide; NYHA, New York Heart Association; SAE, serious adverse event; SGLT2i, sodium–glucose cotransporter 2 inhibitor; SOC, standard of care; SR, sinus rhythm; T1D, type 1 diabetes; VR, visit of randomization; NCT, national clinical trial.

### 7.1 Finerenone Clinical Program

The initial beneficial effects on CV outcomes recorded in recent RCTs in adults with CKD due to T2D or HFmrEF/HFpEF, along with the unmet clinical need not fully addressed by MRAs, have led to further investigation of finerenone. Currently, finerenone is being evaluated in additional large-scale trials in adults with HF with and without CKD to assess its effect on CV death and HF-related events. Table 3 outlines the ongoing phase 3 HF outcome trials with finerenone.

The ongoing MOONRAKER program is designed to extend the findings from the FINEARTS-HF studies to patients hospitalized (or recently discharged) due to HFrEF/HFmrEF/HFpEF or those intolerant/ineligible for steroidal MRA treatment. As a result, this program can help generate evidence for a large patient population, including those who are not eligible for steroidal MRAs [90]. Overall, the MOONRAKER program will identify the efficacy and safety of finerenone across a spectrum of people with HF with or without other CKM conditions. REDEFINE-HF (NCT06008197) is a phase 3 study planned to complete in 2026 assessing the impact of finerenone treatment on the total frequency of HF-related events and CV death across approximately 5200 adults with HFpEF/HFmrEF who are hospitalized with acute decompensated HF [87]. Results from this trial will inform the efficacy and safety of treatment with finerenone in people who are hospitalized (or who have been recently discharged) with acute decompensated HF, a population that commonly has overlapping CKM conditions.

CONFIRMATION-HF (NCT06024746) is a phase 3 study planned to complete in 2025 that is assessing whether early and simultaneous initiation of combination therapy (finerenone and an SGLT2i) will provide a superior clinical benefit (comprising time to death by any cause, number of HF events, time to first HF event, and difference of five or more points on the Kansas City Cardiomyopathy Questionnaire total symptom score) compared with standard of care in 1500 adults hospitalized for HF (or recently discharged) [88]. The results from this trial will be particularly informative as both finerenone and SGLT2is have demonstrated efficacy across CKM subgroups in people with HFmrEF/HFpEF [50, 56, 91, 92]. The study will also provide necessary safety and efficacy data associated with rapid, simultaneous initiation of finerenone and an SGLT2i.

FINALITY-HF (NCT06033950) is a phase 3 study planned to complete in 2028 that is investigating the impact of finerenone on the time to first occurrence of CV death and HF events across approximately 2600 adults with HFrEF who are intolerant/ineligible to receive steroidal MRAs [89].

### 7.2 Investigation of MR Modulators

Currently, other classes of MR-targeting agents are emerging for HF, such as MR modulators. The use of an investigative nonsteroidal MR modulator, balcinrenone, in combination with an SGLT2i is being investigated in the BalanceD-HF (NCT06307652) phase 3 study of 4800 adults with HF (whole-spectrum of LVEF and NYHA class II–IV). The primary composite endpoint is CV death, hospitalization for HF, and an HF event without hospitalization (Table 2) [86].

## 8. Discussion and Conclusions

The prevalence of CKM conditions is rising and the substantial morbidity and mortality associated with this condition will present an increasing management challenge in coming years. MRAs have been established in the management of CV and kidney disease, and their mode of action made this approach a key focus for CKM. The potential clinical advantages have been well demonstrated, with a wealth of clinical evidence demonstrating promising efficacy and safety in the treatment of CKM. The evaluation of the nonsteroidal MRA (finerenone) has also provided additional support for this approach. While hyperkalemia and sexual side effects appear to be of a reduced safety concern with finerenone than with steroidal MRAs, long-term monitoring will be required to evaluate this further. Indeed, long-term efficacy and safety data beyond the duration of a clinical trial will add an important dimension to our understanding, ideally providing insight into real-world use and outcomes. To this end, the ongoing FINE-REAL study is a large, international phase 4 trial evaluating the real-world use of finerenone in the treatment of CKD associated with T2D [93]. This long-term evaluation, initiated in 2022, will continue until 2028, and aims to enrol approximately 5500 participants [94]. Such real-world data are particularly important in the treatment of CKM, as use in patients with complex medical conditions and comorbidities requires careful management and assessment of drug–drug interactions. In addition, real-world data in a diverse population, including the elderly and wider ethnic groups, will be important to ensure efficacy and safety are consistent across all demographics.

While subgroup analysis of trial data has been performed to identify groups that are most likely to benefit from MRA treatment, a greater focus on this is required in order to ensure sufficient data are available to support careful patient selection. Evidence demonstrates that people with HFpEF and those with CKD and T2D benefit from finerenone treatment and studies are ongoing to identify other target populations. As evidence accumulates, incorporation into clinical guidelines will be important to provide direction on the use of MRAs alongside other treatment approaches.

Ongoing clinical trials will investigate the effectiveness of steroidal MRAs and finerenone across a broader spectrum of people with CKM and will provide data to fill the current gaps in our knowledge. The efficacy of finerenone is being investigated across several ongoing large-scale HF RCTs, evaluating its use in people with HF and those who cannot tolerate or are ineligible for steroidal MRA treatment. Ongoing trials of spironolactone and the nonsteroidal MR modulator balcinrenone will also provide insights into their effectiveness in reducing CV-related outcomes and HF-related hospitalizations in patients with HF, addressing gaps from previous studies [86, 95]. These studies are expected to clarify the role of MRAs, both steroidal and nonsteroidal, in the management of CKM and will provide important guidance on therapeutic approaches in this high-risk population.

## Supplementary Material

Supplementary material associated with this article can be found, in the online version, at https://doi.org/10.31083/RCM38690.
