# Supplementary material for: Cardiovascular-Kidney-Metabolic Effects: Steroidal and Nonsteroidal Mineralocorticoid Receptor Antagonists
Source: Rev Cardiovasc Med. 2025 Jul 29;26(7):38690. doi: 10.31083/RCM38690 (PMC12326440; doi:10.31083/RCM38690)
Supplement: Supplementary file 1 [file 2153-8174-26-7-38690-s1.docx]

# Supplementary Material

## Plain Language Summary

Heart failure happens when the heart cannot pump enough blood to meet the body's needs. It is common in people with diabetes, obesity, kidney disease, and high blood pressure. Recent research shows that all these conditions are connected, and doctors refer to this as cardiovascular-kidney-metabolic (CKM) disorder.

A protein on the surface of heart and kidney cells, called the mineralocorticoid receptor, can become overworked through repeated interaction with a hormone called aldosterone. This increases the risk of heart and kidney diseases. Medicines called mineralocorticoid receptor antagonists (MRAs) block this interaction and can improve health outcomes for people with CKM conditions. This review focuses on how MRAs reduce the risk of health problems linked to CKM conditions.

There are several MRAs that can help lower the risk of serious problems such as death or hospital visits in people with CKM conditions. Steroidal MRAs, one type of MRA, are recommended in people with high blood pressure and in people with heart failure. Nonsteroidal MRAs, another type of MRA, have been developed for the treatment of different CKM conditions. Currently, a nonsteroidal MRA is recommended for people with kidney disease and diabetes. Recently, a nonsteroidal MRA has also demonstrated effectiveness in a study with people with heart failure.

Future studies will assess the use of steroidal MRAs in a broader heart failure population, aiming to confirm their benefits and address gaps from earlier studies. Currently, a nonsteroidal MRA is being evaluated in multiple heart failure trials. These trials aim to build on previous findings by including a more diverse group of people with different types of heart failure and CKM conditions.

Overall, MRAs are important for managing heart failure and CKM conditions. Ongoing research will further clarify their benefits and expand treatment options for people with CKM conditions.
